# Case report: Microsatellite instability-high pancreas adenosquamous carcinoma with postoperative liver metastasis recurrence treated with multimodality therapy achieving complete pathological response

**DOI:** 10.3389/fimmu.2024.1456343

**Published:** 2024-12-12

**Authors:** Qinghua Liu, Ruoyun Li, Wei Zhu, Pengfei Zheng

**Affiliations:** ^1^ Department of General Surgery, Lanzhou University Second Hospital, Lanzhou, China; ^2^ Department of Pathology, Lanzhou University Second Hospital, Lanzhou, China

**Keywords:** pancreatic adenosquamous carcinoma, microsatellite instability-high, chemotherapy immunotherapy, targeted therapy, recurrence, metastasis

## Abstract

Pancreatic adenosquamous carcinoma (PASC) is a rare subtype of pancreatic cancer (PC), with no established consensus on the optimal treatment for postoperative liver metastasis recurrence. We report a case of a 48-year-old male patient who underwent radical surgery and was pathologically diagnosed with microsatellite instability-high (MSI-H) PASC. The patient experienced liver metastasis recurrence following single-agent gemcitabine adjuvant chemotherapy. After one session of transarterial chemoembolization (TACE) with oxaliplatin, fluorouracil, and epirubicin, followed by six cycles of adjuvant chemotherapy with gemcitabine and nab-paclitaxel combined with sintilimab immunotherapy and bevacizumab targeted therapy, complete pathological regression of the liver metastasis was achieved. The patient has now reached a 24-month survival period and continues to be monitored at our center. This case illustrates the promise of the proposed treatment regimen, highlighting the significant potential of multimodality strategies in managing metastatic recurrence of MSI-H PASC.

## Introduction

Pancreatic cancer (PC) is one of the most malignant types of cancer, with a 5-year survival rate below 13% and an increasing incidence rate (1). PC consists of various histological types with varying degrees of differentiation, among which pancreatic adenosquamous carcinoma (PASC) is a rare variant, accounting for 1%-4% of PC cases (2). Pathologically, PASC is defined as a mixed tumor originating from the pancreatic duct epithelium, comprising both ductal and squamous differentiation, with at least 30% of the tumor being of the squamous component (3). Because of its unique clinical and histopathological characteristics, PASC exhibits higher malignancy, shorter survival, and a worse prognosis compared to other types of PC (4). Surgical resection is the preferred treatment for PASC, but postoperative metastasis and recurrence frequently occur, significantly affecting the patient prognosis (5). Therefore, the development of effective treatment strategies is crucial for improving the survival rate and quality of life for patients with postoperative metastasis and recurrence of PASC.

Microsatellite instability (MSI) is a genetic trait caused by defective DNA mismatch repair, leading to length variations in microsatellite regions of the genome (6). MSI has become an important biomarker for various malignancies, notably colorectal, endometrial, and gastric cancers, and is correlated with a favorable response to immune checkpoint inhibitors (ICIs) (7). MSI is rare in PC, with an incidence of 1-2% in pancreatic ductal adenocarcinoma (PDAC) (8), there is a paucity of data regarding its prevalence in PASC.

Current treatment protocols for advanced and metastatic or recurrent PC continue to rely predominantly on combinations such as gemcitabine with nab-paclitaxel (GA) or the regimen of 5-fluorouracil, leucovorin, irinotecan, and oxaliplatin (FOLFIRINOX) (9, 10). The GA regimen, when combined with ICIs, has shown a higher disease control rate and longer response duration compared to chemotherapy alone in the treatment of metastatic PC (11, 12). Additionally, bevacizumab, an anti-angiogenic agent, has demonstrated promising results in phase II trials involving patients with metastatic PC (13). Furthermore, Transarterial chemoembolization (TACE) can directly deliver chemotherapeutic agents and embolic materials to the tumor site via the arterial blood supply, proving highly effective in patients with liver metastases (14). At present, the optimal treatment strategy for patients with recurrent liver metastases from microsatellite instability-high (MSI-H) PASC remains uncertain. Consequently, combining different treatment modalities, such as TACE, immunotherapy, and targeted therapy, holds significant promise for creating customized treatment regimens for individual patients. This comprehensive, multimodality treatment strategy has the potential to significantly improve the prognosis of patients with metastatic and recurrent MSI-H PASC. Here, we report a case of MSI-H PASC with liver metastasis recurrence following surgical resection, which achieved complete pathological response after one session of TACE and six cycles of chemotherapy combined with immunotherapy and targeted therapy. This case demonstrated effective treatment with manageable adverse effects, resulting in a satisfactory clinical outcome.

## Case presentation

**Figure 1:**
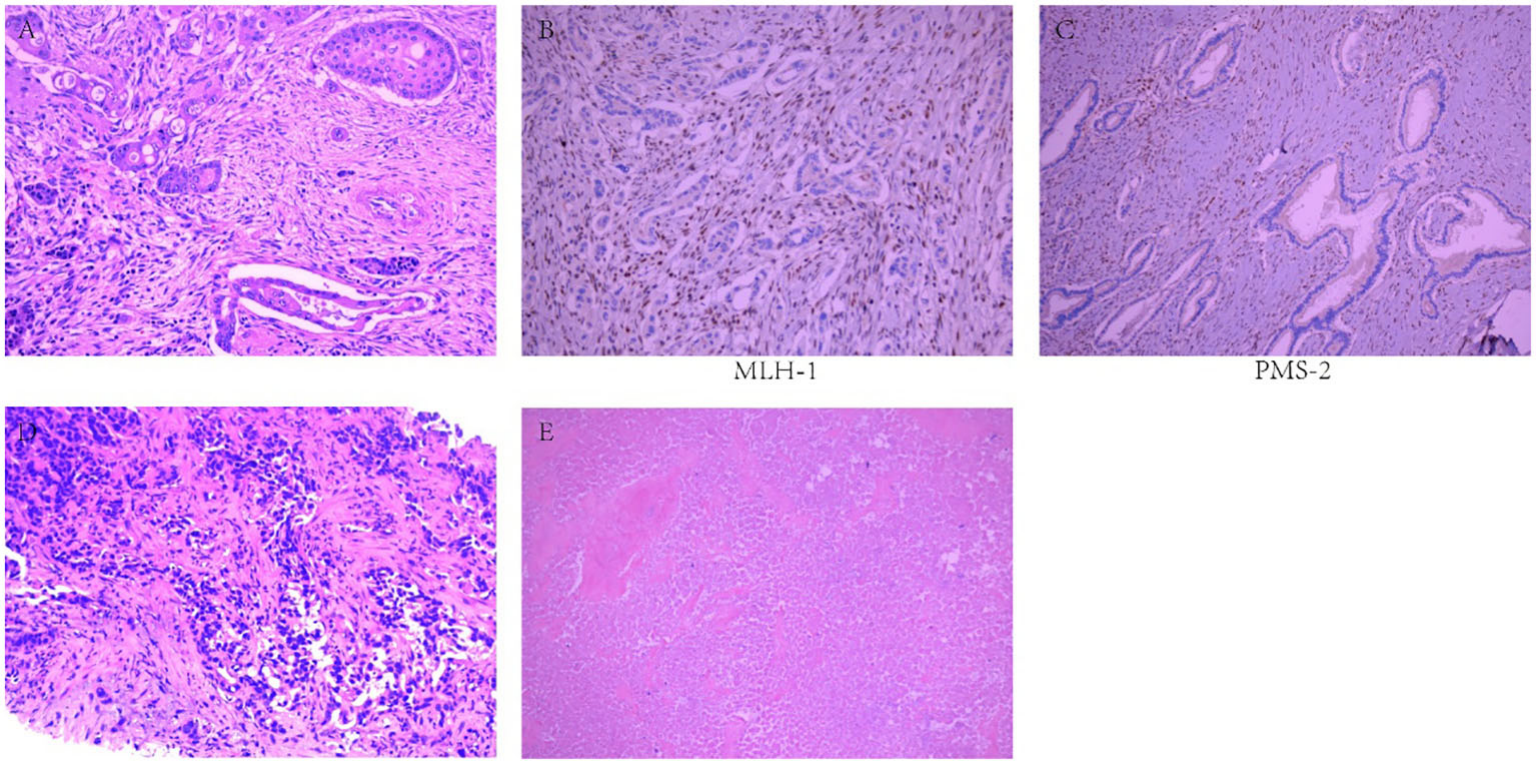
Pathological examination results. **(A)** Moderately to poorly differentiated pancreatic adenosquamous carcinoma. **(B)** MLH1(-). **(C)** PMS2(-). **(D)** Moderately to poorly differentiated adenocarcinoma. **(E)** Necrotic lesions, with no residual tumor cells.

**Figure 2:**
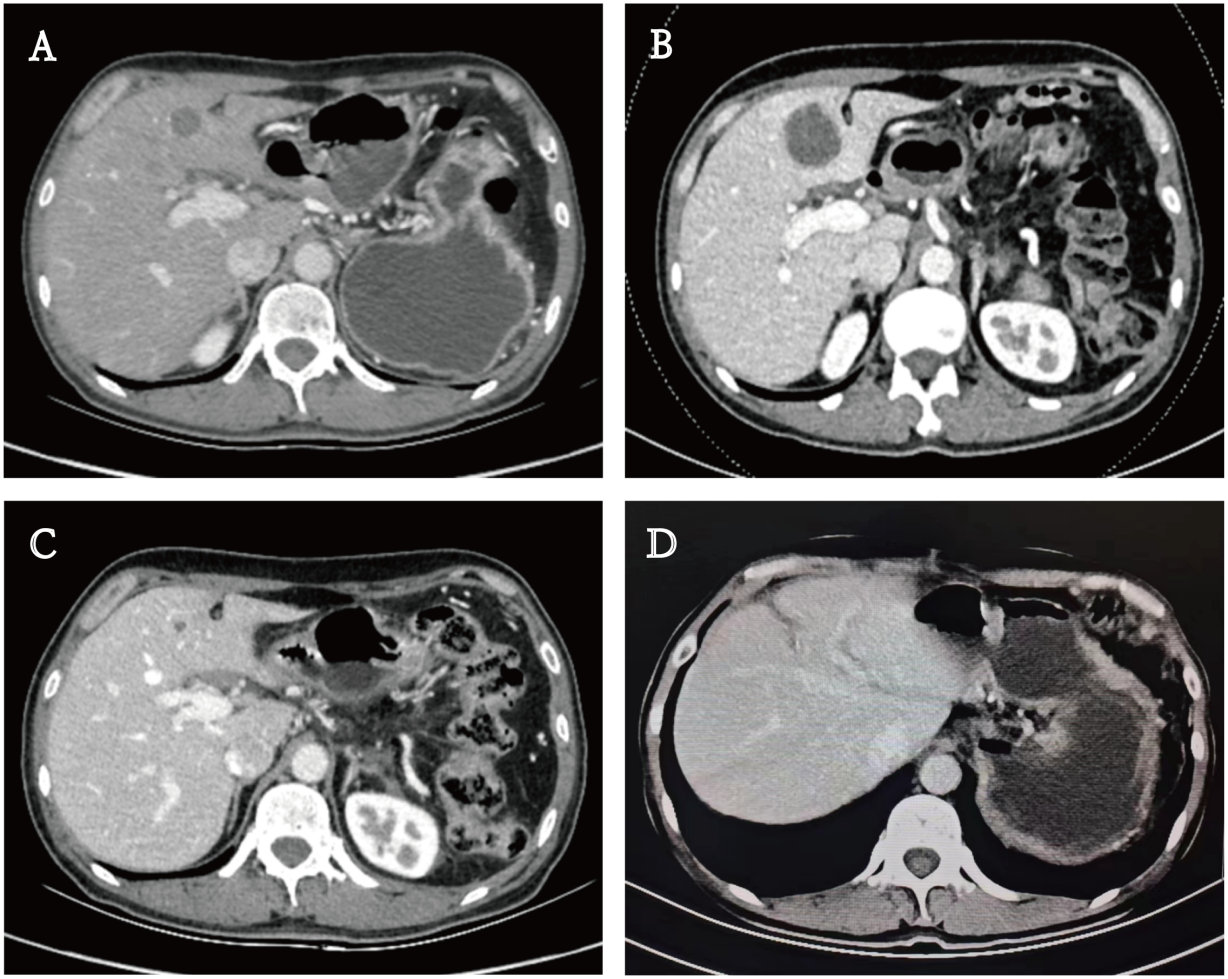
Enhanced computed tomography (CT) scan. **(A)** A low-density lesion in S4 of the liver. **(B, C)** Lesion size changes in the treatment process. **(D)** Imaging at three months postoperatively.

A 48-year-old male patient with PC underwent laparoscopic distal pancreatectomy with splenectomy, followed by six cycles of single-agent gemcitabine adjuvant chemotherapy (1000 mg/m²) at an outside hospital in May 2022. Postoperative pathological findings revealed PASC (**Figure 1A**), moderately to poorly differentiated, clinical stage IIB (T2N1M0). Immunohistochemistry showed MLH1(-) (**Figure 1B**); PMS2(-) (**Figure 1C**); MSH2(+); MSH6(+); P53 (partially positive, suggesting wild-type); Ki-67 (approximately 40% of the squamous carcinoma component and 10% of the adenocarcinoma component); CK19 (+); squamous carcinoma component CK5/6, P63 and P40 (+); adenocarcinoma component CK7 (+). The patient underwent a follow-up examination at our hospital in September 2022. Blood tests showed that CA19-9 was 41.90 U/mL (0-27 U/mL). An enhanced abdominal computed tomography (CT) scan revealed a low-density lesion in segment 4 (S4) of the liver (**Figure 2A**). Further biopsy confirmed the lesion as a moderately to poorly differentiated adenocarcinoma (**Figure 1D**), which, considering the patient’s clinical history and immunohistochemical findings, was attributed to a pancreatic origin. The patient was subsequently diagnosed with MSI-H PASC with postoperative liver metastasis recurrence. The patient’s family history includes both parents having had gastric cancer.

The multidisciplinary team (MDT) evaluated the case, taking into account the patient’s liver metastasis recurrence, MSI-H status, and other potential risks of recurrence. The MDT recommended a treatment regimen comprising chemotherapy combined with immunotherapy and targeted therapy. However, after communicating with the patient and their family, the patient opted to decline the proposed regimen and agreed instead to undergo chemotherapy. Consequently, the patient received adjuvant chemotherapy with gemcitabine (1000 mg/m² on days 1 and 8) combined with nab-paclitaxel (125 mg/m² on days 1 and 8).

In November 2022, the patient returned to our hospital for a follow-up evaluation. The blood test results showed that CA19-9 was 343.00 U/mL (0-27 U/mL), CEA was 85.00 ng/mL (0-3.4 ng/mL), and AFP was 3.35 ng/mL (0-7 ng/mL). CT imaging demonstrated a significant increase in the size of the low-density lesion in S4 of the liver compared to the previous scan (**Figure 2B**). MDT reassessed the case and recommended a treatment regimen consisting of TACE with oxaliplatin (150 mg), fluorouracil (0.75g), and epirubicin (30 mg); gemcitabine (1000 mg/m² on days 1 and 8); nab-paclitaxel (125 mg/m² on days 1 and 8); sintilimab (200 mg); and bevacizumab (200 mg), to be administered in a 21-day cycle. Following a communicating with the patient and their family, they consented to this treatment plan and signed the informed consent forms.

**Figure 3:**
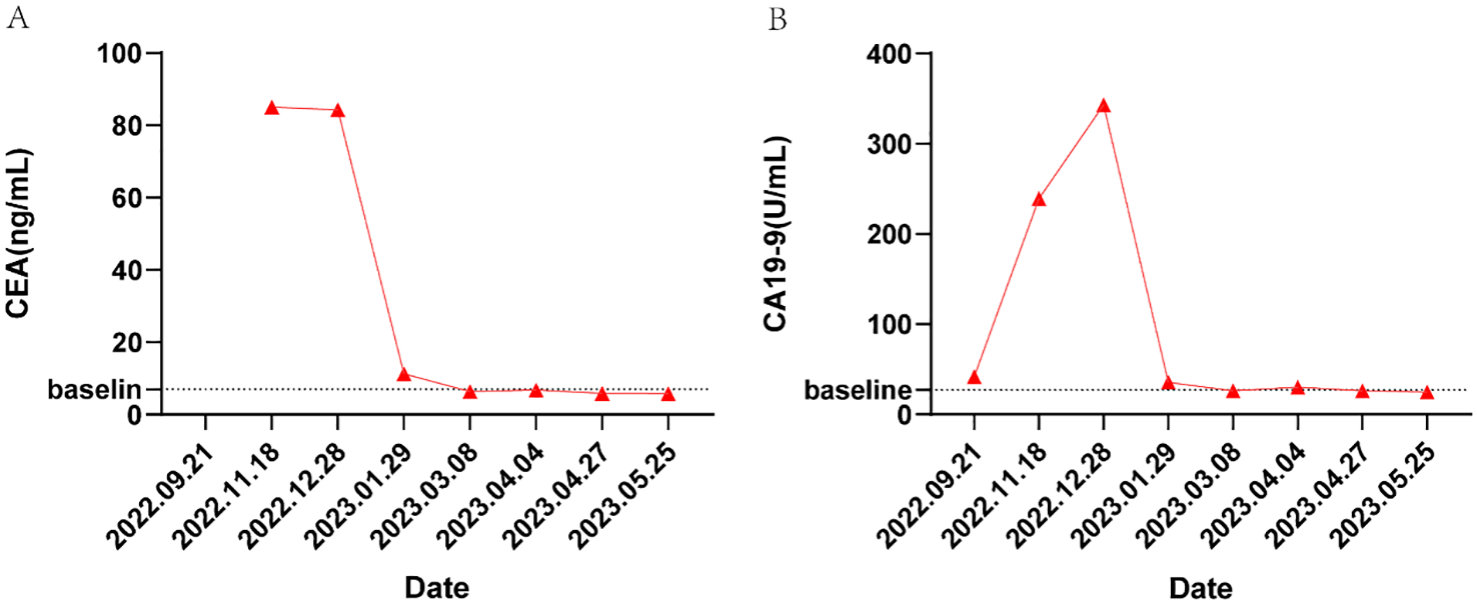
Changes in CEA and CA19-9 value during the disease. **(A)** CEA, **(B)** CA19-9.

In May 2023, the patient had completed one session of TACE and six cycles of multimodality therapy. During treatment, the patient experienced only mild adverse reactions, including nausea, vomiting, and leukopenia, all of which were manageable. Tumor marker levels during the treatment period are illustrated in **Figure 3**. Follow-up CT imaging indicated a reduction in the liver metastases compared to the previous examination (**Figure 2C**), with no evidence of additional recurrence or metastasis. After deliberation by the MDT, surgical intervention was deemed appropriate. Subsequently, the patient underwent laparoscopic partial hepatectomy. Postoperative pathology revealed a 2 cm necrotic nodule in the liver, with no residual tumor cells, minimal fibrosis in the peripheral hepatic vascular bundles, and only mild chronic inflammatory cell infiltration (**Figure 1E**). Complete pathological remission was achieved. The patient was stable during follow-up after surgery, with no abnormalities detected on CT imaging at three months postoperatively (**Figure 2D**).

## Discussion

**Figure 4:**
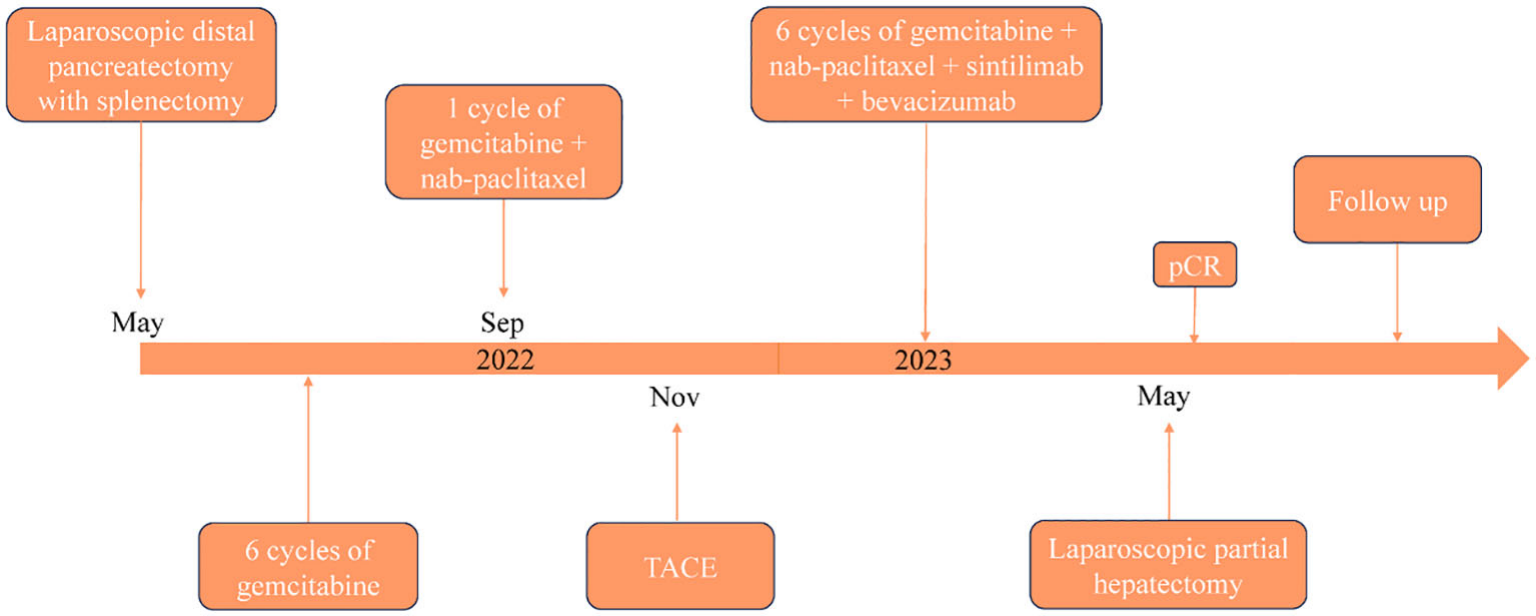
The timeline of the patient’s treatment.

PASC is an exceedingly rare histological subtype of PC, characterized by its pronounced aggressiveness. Despite radical surgery, the prognosis for PASC remains poorer than that for PDAC, with a median survival of approximately 8 months (15). Due to its low incidence, clinical research on PASC has largely been confined to case reports and meta-analyses, with a paucity of large prospective studies to guide clinical treatment strategies (16). Currently, no specific treatment guidelines exist for PASC, however, adjuvant therapy has been shown to significantly extend the median survival in PASC patients. A study reported that the median survival time of patients with PASC receiving platinum-based adjuvant therapy was notably longer than that of those receiving traditional drug-based adjuvant therapy (17). In our case, the patient has adhered to MDT formulated treatment regimen, the patient’s timeline is shown in **Figure 4**, which has extended well beyond the typical median survival period for this disease, with no recurrence or metastasis observed one year after liver metastasis resection. This prolonged survival may be attributable to early surgical intervention and six cycles of multimodality therapy following metastatic recurrence.

The differential diagnosis between PASC and PDAC is crucial due to their similar symptoms, such as weight loss, fatigue, and abdominal discomfort, which can complicate initial diagnosis. Conventional CT or Magnetic resonance imaging features, including a round or lobulated shape, cystic changes, tumor thrombus in the portal vein system, and an annular enhancement pattern, can aid in the differential diagnosis between PASC and PDAC (18, 19). Furthermore, emerging evidence suggests that radiomics analysis may enhance the ability to distinguish between these two tumors (20, 21), which can greatly assist in achieving a definitive diagnosis and guiding follow-up treatment.

MSI-H subtype tumors, a distinct category of neoplasms, are identified in various solid tumors including colorectal, gastric, endometrial, bile duct, liver, and pancreatic cancers (22). MSI-H status serves as a robust biomarker, predicting the efficacy of immunotherapy in many solid tumors. The rarity of MSI-H PASC makes the application of immunotherapy drugs in this subtype limited. Although pembrolizumab has shown good efficacy in patients with PC (23, 24), there are only a few case reports detailing its use in PASC. The high cost of pembrolizumab restricts its accessibility for some patients. In our case, the use of sintilimab demonstrated excellent therapeutic efficacy. The success of sintilimab in this instance aligns with the positive responses observed in other MSI-H malignancies. MSI-H tumors possess a high mutation burden, potentially enhancing neoantigen formation and thereby improving immune recognition and response (25). This suggests that, although MSI-H status is rare in PASC, it may serve as a predictive biomarker for the efficacy of immunotherapy in PASC.

In our case, the patient’s immunohistochemistry showed MLH1(-) and PMS2(-), both of the patient’s parents had gastric cancer. We considered the possibility of Lynch syndrome (LS) and suggested that the patient undergo genetic testing to clarify the diagnosis. However, the patient and his family declined the suggestion because of the high cost. LS is a genetic disorder caused by mutations in the DNA mismatch repair genes, which can predispose an individual to a variety of cancers. Screening and genetic counseling play a vital role in the management of LS. Screening typically starts with identifying individuals at risk, often based on family history or clinical features such as early-onset colorectal cancer or a history of related cancers. Individuals with LS should undergo colonoscopy every 1 to 2 years to screen for colorectal cancer and precancerous polyps. Depending on family history, screening for other LS-associated cancers (such as gastric, pancreatic, or urinary tract cancers) may also be recommended (26). Genetic counseling helps affected individuals understand their condition, the implications for family members, and options for cancer surveillance and prevention. It is essential for at-risk relatives to consider predictive genetic testing, which enables preventive measures and informed lifestyle decisions (27). Studies have shown that people with LS have approximately a 9-fold higher risk of developing PC than the general population. LS plays an important role in early screening for PC and in exploring personalized treatment options such as immunotherapy (28). Early screening may improve prognosis in high-risk populations. Studies suggest that patients with one or more first-degree relatives with PC and LS should be monitored regularly with magnetic resonance imaging and ultrasound endoscopy (29). Future studies should further refine the understanding of the role of LS in PC, leading to better outcomes for patients.

In recent years, immunotherapy has emerged as a pivotal area in cancer treatment, demonstrating substantial therapeutic benefits across various solid tumors and effectively extending patient survival. However, progress in immunotherapy for PC has been limited. The efficacy of ICIs in PC is generally poor compared to other malignancies, primarily because the immunosuppressive microenvironment characteristic of this cancer, driven by oncogenic KRAS mutations, disruption of innate and adaptive anti-cancer immunity, and constraints on T-cell immune response initiation, resulting in significant immune resistance (30). Nevertheless, ICIs have shown some progress in the treatment of MSI-H PC. One study demonstrated the preferential use of ICIs over cytotoxic chemotherapy in patients with MSI-H PC requiring systemic therapy, including metastatic and adjuvant/neoadjuvant treatment (31). Another study showed that ICIs were effective and well tolerated in patients with advanced MSI PC (32). Because of the low incidence of PASC, there is currently no standardized immunotherapy regimen. Lee et al (33). found no significant difference in survival rates between PD-L1 positive and PD-L1 negative PASC patients, possibly reflecting pancreatic cancer’s insensitivity to monotherapy with ICIs. Developing novel ICIs and integrating them with surgery and adjuvant therapy may enhance survival in PASC patients.

Anti-angiogenic agents have shown promise in PC treatment (34). A phase III clinical trial demonstrated that in 301 patients with PDAC, the combination of gemcitabine and erlotinib with bevacizumab significantly improved progression-free survival, although no significant extension in overall survival was observed (35). Multiple clinical studies have indicated that bevacizumab is efficacious in treating hepatocellular carcinoma (36, 37). Based on these findings, we administered bevacizumab to our patient, achieving positive results, thereby providing which may serve as a reference for subsequent targeted therapy in PASC treatment.

However, our case report has several limitations. First, the findings are based on a single patient, which restricts the generalizability of the results. Furthermore, considering tumor heterogeneity and individual variations in patient health status and treatment response, the success observed in this case may not be replicable in all MSI-H PASC patients. Finally, the MSI status of the patient was determined via immunohistochemistry without confirmation by polymerase chain reaction (PCR) or high-throughput sequencing, which may introduce potential inaccuracies. Future studies with larger cohorts or a series of cases are necessary to validate these findings and provide a more precise assessment of MSI status, thereby generating more reliable data on the efficacy of multimodality therapy.

## Conclusion

In conclusion, the multimodality treatment approach combining TACE, chemotherapy, sintilimab and bevacizumab in this case offers a promising example and valuable insights for managing MSI-H PASC with metastatic recurrence following first-line treatment. This case underscores the utility of MSI in guiding therapeutic decisions and highlights the need for further research and clinical trials to optimize treatment regimens and improve outcomes in this challenging malignancy.

## Data Availability

The raw data supporting the conclusions of this article will be made available by the authors, without undue reservation.
